# The effects of physical activity on brain structure and neurophysiological functioning in children: A systematic review and meta-analysis

**DOI:** 10.1016/j.dcn.2020.100828

**Published:** 2020-07-25

**Authors:** Anna Meijer, Marsh Königs, Gerben T. Vermeulen, Chris Visscher, Roel J. Bosker, Esther Hartman, Jaap Oosterlaan

**Affiliations:** a Vrije Universiteit, Clinical Neuropsychology Section, Amsterdam, The Netherlands; b Emma Children’s Hospital, Amsterdam UMC, University of Amsterdam, Emma Neuroscience Group, Department of Pediatrics, Amsterdam Reproduction & Development, Amsterdam, The Netherlands; c University of Groningen, Groningen Institute for Educational Research, Groningen, The Netherlands; d University of Groningen, University Medical Center Groningen, Center for Human Movement Sciences, Groningen, The Netherlands

**Keywords:** Neuroimaging, Magnetic resonance imaging, Electroencephalography, Physical activity, Children, Systematic review, Meta-analysis

## Abstract

This study is the first to systematically review and quantify the effects of physical activity on brain structure and neurophysiological functioning in children. Electronic data bases were searched for relevant studies. Studies that met the following criteria were included: (1) used an RCT or cross-over design, (2) examined the effects of physical activity on brain structure and/or neurophysiological functioning, (3) included children (5–12 years old) (4) included a control group (RCTs) or control condition (cross-over trials). A total of 26 and 20 studies were included in the systematic review and meta-analysis, respectively, representing and accompanying 973 and 782 unique children. Main analyses were separated for short-term and long-term physical activity and for effects on brain structure and neurophysiological functioning with a distinction between children from healthy and clinical populations. We found evidence for significant beneficial effects of long-term physical activity on neurophysiological functioning (*d* = 0.39, *p* < 0.001). In addition, short-term physical activity may induce changes in neurophysiological functioning (*d* = 0.32, *p* = 0.044), although this evidence showed limited robustness. No meta-analytic evidence was found for positive effects on brain structure. The results underline the importance of physical activity for brain development in children.

## 1 Introduction

Physical activity has been associated with a range of physical, behavioral, cognitive and academic benefits (Álvarez-Bueno et al., 2017; de Greeff et al., 2018; Hillman et al., 2008; Penedo and Dahn, 2005; Warburton et al., 2006). A growing body of literature indicates that the majority of the pediatric population comes not even close to the recommended 60 min of moderate intense physical activity per day for children (Verloigne et al., 2012). Moreover, the prevalence of a sedentary lifestyle among children is rapidly increasing (Gabel et al., 2016; Tremblay et al., 2011).

The evident lack of physical activity among children is especially worrisome in the light of existing evidence on beneficial effects of physical activity on brain development (Van Praag et al., 1999). The beneficial effects of physical activity on the brain are thought to have more long-lasting effects in childhood as compared to adulthood, suggesting that physical activity in childhood also contributes to brain functioning in adult life (Hopkins et al., 2011; Luna, 2009). In line with this idea, physical activity is also suggested as a potential treatment to improve brain development in pediatric clinical populations, such as children with depression or Attention Deficit Hyperactivity Disorder (ADHD) (Brown et al., 2013; Smith et al., 2013). For example, exercise intervention studies indicated beneficial effects on behavioral and cognitive symptoms of ADHD (Ng et al., 2017; Suarez-Manzano et al., 2018). Also, altered brain function and cognitive dysfunction have been found in obese children compared with leaner children (Bruce et al., 2011; Yau et al., 2012). Recent studies have shown that exercise also has beneficial effects on cognition in this population (Veronese et al., 2017). Nevertheless, to this date it remains largely unknown which underlying neural mechanisms give rise to the beneficial effects of physical activity in children.

Findings from fundamental neuroscience have identified several pathways through which physical activity may act on brain structure and neurophysiological functioning. A single bout of physical activity (or short-term physical activity) has been shown to directly enhance cerebral blood flow (Querido and Sheel, 2007) and to trigger the upregulation of neurotransmitters that facilitate cognitive processes (e.g. epinephrine, dopamine; (Dishman et al., 2006; McAuley et al., 2004). These immediate effects resulting from a single bout of physical activity are often referred to as acute effects. Longer periods of continuous physical activity (long-term physical activity) are thought to trigger additional pathways that exert beneficial effects on brain development. Long-term physical activity has been shown to elevate the levels of neurotropic factors (e.g. brain-derived neurotropic factor and nerve growth factor), which are known to boost neural blood vessel formation and neurogenesis (Colcombe et al., 2006; Dishman et al., 2006; Swain et al., 2003). These prolonged effects of long-term physical activity are often referred to as chronic effects. The observed acute and chronic effects indicate that physical activity is potent to change brain structure and neurophysiological functioning through differential mechanisms.

In line with this evidence, previous studies in children have revealed associations between physical fitness - which is considered as an indirect measure of long-term physical activity (Caspersen et al., 1985) - and brain structure as well as neurophysiological function. Regarding brain structure for example, cross-sectional magnetic resonance imaging (MRI) studies in 9−10-year-old children have shown that higher aerobic fitness is associated with larger brain volumes, including volumes of the basal ganglia and the bilateral hippocampus (Chaddock et al., 2010a, 2010b). Regarding neurophysiological functioning, a number of cross-sectional electroencephalography (EEG) studies in 9−10 year old children has shown that higher aerobic fitness is associated with greater allocation of attentional resources (as measured by the P3 component of the event-related potential) on tasks measuring interference control (Chaddock et al., 2012; Pontifex et al., 2011), cognitive flexibility (Pontifex et al., 2011), language processing (Scudder et al., 2014) and mathematical processing (Moore et al., 2014). Although these cross-sectional studies indicate an association between physical fitness and neural mechanisms and support the idea that long-term physical activity has beneficial effects on the child’s brain, these association studies do not provide causal evidence. Instead, intervention effectiveness studies, such as randomized controlled trials (RCTs) and cross-over trials, are necessary to evaluate causal effects.

The current study aims to provide an overview of all available RCTs and cross-over trials testing the causal effects of physical activity on brain structure and neurophysiological functioning in children. Earlier reviews of studies on this topic did not use a systematic approach (Chaddock et al., 2011), or conclusions were (partly) based on studies using study designs that cannot provide evidence on causation (association studies or quasi-experimental designs) or did no attempt to quantify the effects (Donnelly et al., 2016; Valkenborghs et al., 2019). The mechanisms underlying the effects of physical activity on neuroimaging measures may be influenced by health status. Therefore, the current review and meta-analysis will make a distinction between studies in healthy and clinical samples of children. Changes in brain structure and neurophysiological functioning paralleled by changes in cognitive functioning potentially provide more insight into the mechanisms underlying the effects of physical activity. Hence, we provide a narrative review in which we determine whether reported changes in brain structure and neurophysiological functioning are accompanied by beneficial effects of physical activity on cognitive functioning, as reported by a correlation/regression analysis or coinciding positive effects of physical on neuroimaging and behavioral outcome measures. Where possible, we will quantify the magnitude of the effect of physical activity on brain structure and neurophysiological functioning using meta-analytic methods.

## 2 Methods

### 2.1 Study selection

This systematic review and meta-analysis included empirical studies that: (1) used an RCT or cross-over design, (2) examined the effects of moderate to vigorous physical activity on brain structure and/or neurophysiological functioning, where moderate to vigorous physical activity was defined as physical activity that requires a moderate amount of effort and noticeably accelerates the heart rate (World Health Organization, 2010), (3) included children with an average age between 5–12 years old (4) and included a no intervention control group (RCTs) or control condition (cross-over trials).

The electronic databases PubMed, Embase, SportDiscus and Cochrane Library were searched combining search terms (MeSH and thesaurus terms) related to *physical exercise* and *children,* and *Brain Imaging or electroencephalography* and their equivalents (Table 1A, see Appendix; last search December 2019). The reference lists of all included articles were manually searched for additional relevant articles.

**Fig. 1:**
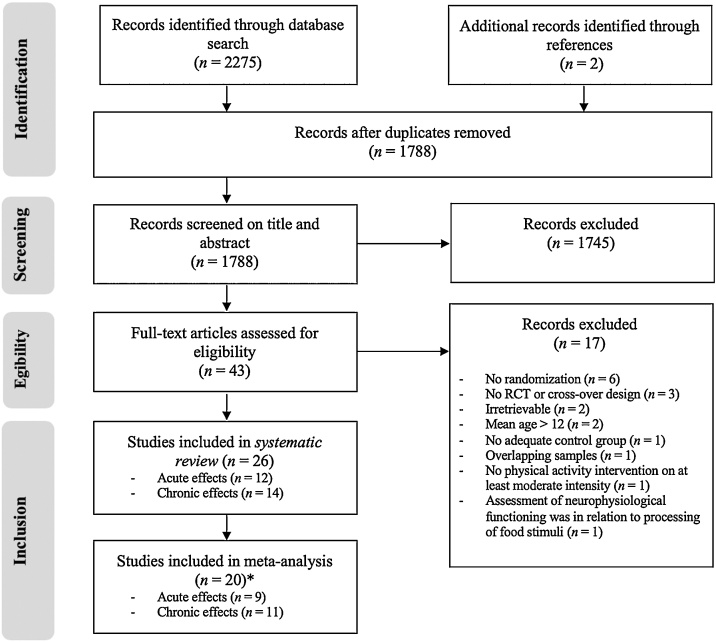
PRISMA flow diagram of studies through the review process *Not all studies were included in the meta-analysis because these studies did not report sufficient information.

This systematic review and meta-analysis was performed according to PRISMA guidelines (Moher et al., 2009). The article identification, screening and selection process was performed by two independent reviewers (AM + GV, Fig. 1). The initial search retrieved 2275 unique articles, of which 37 articles were deemed relevant based on the screening of title and abstract. These 37 articles were further assessed for eligibility based on full-texts, after which 23 articles met all inclusion criteria. Two studies were excluded because of contaminating factors such as no sufficient intensity of the physical activity intervention (relaxation; Chan et al., 2015) or the assessment of neurophysiological functioning in relation to the processing of food stimuli (Masterson et al., 2018). Finally, a total of 26 articles was included in the narrative review, of which 20 articles were suitable for meta-analysis.

### 2.2 Data extraction

The following data were extracted from the included articles: (1) sample characteristics (for each study group: sample size, mean age and sex distribution); (2) intervention or control features (type, intensity and frequency of physical activity or control sessions); (3) outcome measures (imaging modality and cognitive tests assessed, if available).

### 2.3 Risk of bias assessment

The quality of included studies was independently assessed by two authors (AM + GV) using the Cochrane Collaboration’s tool for risk of bias in randomized trials (Higgins et al., 2011). This tool examines selection bias (random sequence generation and allocation concealment), performance bias (blinding participants and personnel), detection bias (blinding of outcome assessment), attrition bias (participants lost during study) and reporting bias (selective outcome reporting of prespecified outcome measures in methods sections or clinical trial registers). In addition, we evaluated all studies on sampling bias (representativeness of the sample for the targeted pediatric population). For each of these bias categories, studies were classified into low, unclear, or high risk of bias. Inter-rater discrepancies were resolved by consensus.

### 2.4 Statistical analysis

Statistical analysis was performed using Comprehensive Meta-Analysis Software (CMA) - version 3 (Borenstein et al., 2013). Meta-analytic effect sizes were calculated separately for acute and chronic effects of physical activity. Further distinction was made between studies concerning (1) brain structure and (2) neurophysiological functioning, with further distinction between (3) children from healthy and (4) clinical samples. In addition, separate meta-analytic effects were calculated for subgroups of studies that reported identical outcome measures in at least two studies (e.g. P3 amplitude, P3 latency).

To calculate meta-analytic effect sizes, individual studies’ effect sizes pertaining to each outcome measure were calculated using statistics describing the interaction effect between group and time for RCTs and between condition and session for cross-over trials (n, standard deviation, F value, p-value or the pre/post mean and standard deviation). Beneficial effects of physical activity on outcome measures (in the experimental group/condition as compared to the control group/condition) were expressed as positive effect sizes. Interpretation of neuroimaging measures is not always straightforward (de-Wit et al., 2016). Therefore, interpretation of the individual studies’ effect sizes (positive vs. negative) was based on the following sequential decision chain: (1) the interpretation of the authors, if supported by empirical evidence, (2) the direction of related cognitive effects, or (3) empirical evidence on the developmental course of the outcome measure, in which effects in the maturation direction were interpreted as positive. This decision chain did not result in a clear interpretation for functional MRI measures (fMRI; *k* = 3). Increased fMRI activation (as measured by the Blood Oxygen Level-Dependent [BOLD] signal) during cognitive tasks (active-state fMRI) could be interpreted as a reflection of greater flexible processing, but decreased activation could also be interpreted as improved learning and an efficiency effect (Jansma et al., 2001). The effect size of these three studies were, according to the authors’ interpretation, initially labeled as positive. The meta-analytic results that included these studies were only interpreted in terms of changes in neurophysiological functioning, i.e. no conclusions were made about the direction of the results (beneficial or detrimental). To determine the valence of the meta-analytic effects, we performed a sensitivity analysis by means of additional meta-analysis leaving out the fMRI findings.”

To prevent inflation of homogeneity due to correlated observations, effect sizes of multiple outcome measures from the same study were averaged before calculation of the meta-analytic effect sizes (Borenstein et al., 2009). Meta-analytic effect sizes were calculated using the random model to correct for heterogeneity between studies introduced by differences in experimental design, measurement modality and analytic design. The derived meta-analytic effect sizes were further weighted by the study inverse variance, thereby accounting for sample size and measurement error (Borenstein et al., 2009). Meta-analytic effects were interpreted using Cohen’s guidelines, including definitions of small (*d* = 0.2−0.5), moderate (*d* = 0.5−0.7) and large effect sizes (*d* > 0.7; Cohen, 1988).

Heterogeneity of eﬀ ;ect sizes was assessed using the *I*^2^ statistic, where values of 25 %, 50 % and 75 % were indicative of low, moderate and high heterogeneity, respectively (Higgins and Thompson, 2002). As the *I*^2^ statistic can be biased in meta-analyses with small samples, confidence intervals for each effect size were included (von Hippel, 2015). Meta-analytic effect sizes were subjected to analyses of robustness (leave-one-out analysis and p-curve analysis) and the possibility of publication bias (Rosenthal’s fail-safe *n*, Egger funnel plot asymmetry and the test of excessive significance). The leave-one-out method was used to check the influence of single studies by iteratively removing each individual study from the calculation of the meta-analytic effect sizes. We explored whether meta-analytic effects where disproportionately driven by a single study by visual interpretation of the leave-one-out forest plots in combination with the statistical results (Viechtbauer and Cheung, 2010). P-curve analysis was performed to check whether the distribution of effect sizes that contribute to a significant meta-analytic effect size is indicative of a true effect (i.e. the meta-analytic effect has evidential value; Simonsohn et al., 2015). P-curves were created using the p-curve application (http://www.p-curve.com/app4, 2018), where all p-values below .05 are plotted on the x-axis and the percentage of studies yielding these p-values are plotted on the y-axis. According to Simonsohn et al. (2015), (1) right skewed p-curves are indicative of evidential value, as true effects tend to be highly significant, (2) flat p-curves indicate no evidential value and (3) left skewed p-curves are indicative of flexibility in data-analysis (p-hacking), as these contain many p-values just below .05. Evidential value in a set of studies requires that the curve for p-values lower than .025 (i.e. the half p-curve), is significantly right skewed (p-value test for right skew <.05), or that the p-value of this skewness test is at p < .10 for both the full and half p-curve (Simonsohn et al., 2015). In case the results indicate no evidential value, a follow-up test is performed to test whether the set of studies had insufficient power to detect evidential value (Simonsohn et al., 2015). If this test is significant (p < .05), the conclusion is that the set of studies does not have sufficient analytic power to detect evidential value. Publication bias was assessed by Egger funnel plot asymmetry (Egger et al., 1997), the Rosenthal’s fail-safe *n* (Rosenthal, 1995) and the test of excessive significance (Ioannidis and Trikalinos, 2007). Rosenthal’s fail-safe *n* was calculated to determine the necessary number of studies to nullify the overall effect. Fail-safe n values > 5k + 10 were considered robust, where k refers to the number of samples on which the relevant effect size was calculated. Furthermore, the test of excessive significance (Ioannidis and Trikalinos, 2007) was used to compare the observed number of significant studies to the expected number of significant studies with a χ^2^-test (*p* < .10). The expected number was based on the estimated overall effect-size and the power to detect this effect in the individual studies. All tests of significance were two-sided with α = .05.

## 3 Results

This results section provides a narrative description of the findings of all studies (*k* = 26), followed by the meta-analytic findings (*k* = 20). We first distinguish between studies addressing acute and chronic effects. Further distinction is made between (1) studies focusing on brain structure and neurophysiological functioning and (2) studies in healthy and clinical samples. The main study characteristics are presented in Table 1 (acute effects) and Table 2 (chronic effects). An explanation and interpretation of the effect direction of all imaging measures is presented in Table 3.

**Table 1:** Summary of study characteristics and results of studies including acute effects of short-term physical activity programs.

Study	Sample	Design and assessment	Intervention	Mean Age	*N* (% male)	Duration (min)	Outcome modality	Outcome measurements	Significant differences (>0.05) between physical activity and control sessions
Chen et al. (2016)	Healthy children	Cross-over + post-intervention	- Ergometer cycling (60−69% HRmax) - Sedentary rest session	10	9 (55 %)	35	Brain function (MRI)	- Active-state fMRI: Left parietal cortex - Active-state fMRI: Right parietal cortex - Active-state fMRI: Left hippocampus - Active-state fMRI: Left cerebellum - Active-state fMRI: Right cerebellum *- Working memory RT (N-back task)* *- Working memory accuracy (N-back task)*	> Increased activation after PA session* > Increased activation after PA session* > Increased activation after PA session* > Increased activation after PA session* > Increased activation after PA session* > Shorter RT (only in the 2-back condition) after the PA session *> ns*
Chu et al. (2017)	Healthy children	Cross-over + post- intervention	- Treadmill running (65−75 HRR) - Reading session	10.5	20 (100 %)	30	Brain function (EEG)	- P3 amplitude - Conflict SP amplitude - *Reaction time (Stroop task)* *- Accuracy (Stroop task)*	> Greater P3 amplitudes after PA session > Smaller conflict SP amplitudes after the PA session* > Marginally shorter RT after the PA session (p = 0.057) *> ns*
Chuang et al. (2015)	ADHD	Cross-over + pre/post - intervention	- Treadmill running (60 % HRR) - Video-watching	9.4	19 (84 %)	30	Brain function (EEG)	- CNV 1 amplitude - CNV 2 amplitude *- Motor inhibition RT (Go/No Go task)* *- Motor inhibition accuracy (Go/No Go task)*	> ns > Smaller frontal CNV 2 amplitudes for No Go Stimuli after the PA condition *> Shorter RT after the PA session* *> ns*
Hillman et al. (2009)	Healthy children	Cross-over + post - intervention	- Treadmill running (60 % HRmax) - Rest session	9.6	20 (60 %)	20	Brain function (EEG)	- P3 amplitude - P3 latency *- Inhibitory control RT (Flanker task)* *- Inhibitory control accuracy (Flanker task)* *- Reading comprehension (WRAT3)* *- Arithmetic (WRAT3)* *- Spelling (WRAT3)*	> Greater P3 amplitudes after the PA session* > ns* > ns *> Improvement after the PA session* *> Improvement after the PA session* *> ns* *> ns*
Huang et al. (2018)	ADHD	Cross-over + pre/post - intervention	- Treadmill running (65−75% HRR) - Video-watching	9.5	24 (100 %)	30	Brain function (EEG)	- Resting-state: Alpha power - Resting-state: Beta power - Resting-state: Theta power - Resting-state: Theta/alpha ratio - Resting-state: Theta/beta ratio	> ns* > ns* > ns* > ns* > Lower theta/beta ratio after the PA condition*
Hung et al. (2016)	ADHD	Cross-over + post - intervention	- Treadmill running (50−70% HRR) - Video-watching	10.2	34 (97 %)	30	Brain function (EEG)	- P3 amplitude (Global switching paradigm) - P3 amplitude (Local switching paradigm) - P3 latency (Global switching paradigm) - P3 latency (Local switching paradigm) *- Working memory RT (Global switching paradigm)* *- Working memory accuracy (Global switching paradigm)* *- Inhibition RT (Local switching paradigm)* *- Inhibition accuracy (Local switching paradigm)*	> Greater P3 amplitudes during the trials that required working memory involvement after the PA session* > ns* > ns* > ns* > ns *> ns* *> ns* *> ns*
Lind et al. (2019)	Healthy	Cross-over + pre/post - intervention	- Football (70 – 100% HRR) - Walking football (60−80% HHR) - Football video watching group	11.8	17 (n/a) 16 (n/a) 16 (n/a)	20	Brain function (EEG)	- P3 amplitude - P3 latency *- Inhibitory control RT (Flanker task)* *- Inhibitory control accuracy (Flanker task)* *- Declarative memory (Visual memory task)*	> Greater P3 amplitudes at Fz in children performing football compared to walking football group and the control group* > ns* > *Shorter RT the football condition compared to the walking football group* > *ns* > *ns*
Mierau et al. (2014)	Healthy children	Cross-over + pre/post - intervention	- Movement games + soccer match (HR is not reported) - Rest session	5.8	10 (100 %)	45	Brain function (EEG)	- Alpha-1 power (resting-state) - Beta-1 power (resting-state) - Beta-2 power (resting-state) - Alpha-1 power (active-state) - Beta-1 power (active-state) - Beta-2 power (active-state) *- Attention RT (Determination test)* *- Attention accuracy (Determination test)*	> Increased alpha-1 power after the PA session > Decreased beta-1 power at frontal sites after the PA session > Decreased beta-2 power at frontal sites after the PA session > ns > Decreased beta-1 power at frontal sites after the PA session > Decreased beta-2 power at frontal and central sites after the PA session *> ns* *> ns*
Pontifex et al. (2013)	- ADHD (n = 20) - Healthy children (n = 20)	Cross-over + post - intervention	- Treadmill running 65−75% HRmax - Sedentary reading session	9.8	40 (70 %)	20	Brain function (EEG)	- P3 amplitude - P3 latency - ERN *- Inhibitory control RT (Flanker task)* *- Inhibitory control accuracy (Flanker task)* *- Reading comprehension (WRAT3)* *- Arithmetic (WRAT3)* *- Spelling (WRAT3)*	> Greater P3 amplitudes after the PA session (ADHD + healthy children)* > Shorter P3 latencies after the PA session (ADHD + healthy children)* > ns *> ns* *> Improvement in acc. after PA session (ADHD + healthy children)* *> Improvement in reading after PA session (ADHD + healthy children)* *> Improvement in arithmetic after PA session (ADHD + healthy children)* *> ns*
Pontifex et al. (2018)	Healthy children	Cross-over + pre/post - intervention	- Treadmill running 70 % HRmax - Treadmill walking on the lowest possible intensity	10.2	41 (56 %)	20	Cerebral blood flow (MRI)	> Cerebral blood flow	> ns
St Louis Deschenes et al. (2015)	Healthy children	Cross-over + post - intervention	-Ergometer cycling (50 % HRmax) -Sedentary session on ergometer	10.4	32 (44 %)	30	Brain function (EEG)	- P3a amplitude - P3a latency - P3b amplitude - P3b latency *- Attention accuracy (Oddball task)* *- Attention RT (Oddball task)*	> ns > ns > Greater P3b amplitudes after the PA session* > ns *> ns* *> ns*
Wollseiffen et al. (2018)	Healthy children	Cross-over + pre/post - intervention	- Aerobic exercise (HR is not reported) - Art class	8−10 years	16 (50 %)	45	Brain function (EEG)	- Cortical current density activity *- Education attainment (VERA-3)* *- Working memory (MemoryMatrix)*	> Decreased activity after the PA session* > ns *> ns*

Note. *, Measures are included in meta-analysis. The measures without an asterisk were not included in further meta-analysis because additional information was not available; Italicized measures are behavioral measures.

ADHD, Attention deficit hyperactivity disorder; CNV, contingent negative variation; CRN, Correct-related negativity; EEG, Electroencephalography; ERN, Error-related negativity; HR, heart rate; HRmax, predicted maximal heart rate; HRR, heart rate reserve; MRI, magnetic resonance imaging; ns, not significant; PA, physical activity; RT, reaction time; WRAT3, Wide range Achievement Test 3rd edition.

**Table 2:** Summary of study characteristics and results of studies including chronic effects of long-term physical activity.

Study	Sample	Design and assessment	Intervention	Mean Age	*N* (% male)	Duration (min.)	Outcome modality	Outcome measurements	Significant differences (>0.05) between physical activity and control sessions
Chaddock-Heyman et al. (2013)	Healthy children	RCT + pre/post - intervention	-FITKIDS physical activity program -Wait-list control group	8.9 8.9	14 (50 %)9 (66 %)	600 p/w 36 weeks	Brain function (MRI)	- Active-state fMRI: Anterior PFC - Active-state fMRI: Anterior cingulate cortex *- Inhibitory control RT (Flanker task)* *- Inhibitory control accuracy (Flanker task)*	> Decreased activation in PA condition* > ns* > Within PA condition improvement during incongruent and neutral trials *> Within PA condition improvement during neutral trials*
Chaddock-Heyman et al. (2018)	Healthy children	RCT + pre/post - intervention	- FITKIDS2 physical activity program -Wait-list control group	8.7 8.7	76 (49 %)67 (49 %)	600 p/w 36 weeks	Brain structure (DTI)	- WMI FA - WMI AD - WMI RD	> Increased FA in the PA condition in: gCC* > ns* > Decreased RD in the PA condition in: gCC*
Davis et al. (2011)	Obese children	RCT + pre/post - intervention	- Aerobic physical activity program - Control group	9.6 9.6	10 (n/a) 9 (n/a)	100 or 200 p/w 13 weeks	Brain function (MRI)	- Active-state fMRI: FEF - Active-state fMRI: SEF - Active-state fMRI: PFC - Active-state fMRI: PPC *- Inhibition (Antisaccade task)*	> ns > ns > Increased activation in bilateral PFC in PA condition* > Decreased activation in bilateral PPC in PA condition* > Not reported
Drollette et al. (2018)^A^	Healthy children	RCT + pre/post - intervention	- FITKIDS2 physical activity program -Wait-list control group	8.7 8.8	139 (51 %)169 (51 %)	600 p/w 36 weeks	Brain function (EEG)	- CRN - ERN *- Attention inhibition RT (Flanker task)* *- Attention inhibition accuracy (Flanker task)*	> ns > Increased ERN amplitudes in the control condition* > ns *> ns*
Hillman et al. (2014)^A^	Healthy children	RCT + pre/post - intervention	- FITTKIDS physical activity program -Wait-list control group	8.8 8.8	109 (51 %) 112 (46 %)	600 p/w 36 weeks	Brain function (EEG)	- P3 amplitude (Flanker task) - P3 latency (Flanker task) - P3 amplitude (Switch task) - P3 latency (Switch task) *- Attention inhibition RT (Flanker task)* *- Attention inhibition accuracy (Flanker task)* *- Cognitive flexibility RT (Switch task)* *- Cognitive flexibility accuracy (Switch task)*	> Greater P3 amplitudes in the PA condition for incongruent trials* > Shorter P3 latencies in the PA condition for incongruent trials* > Greater P3 amplitudes in the PA condition for heterogeneous trials* > ns* > ns *> Greater improvement in PA condition* *> ns* *> Greater improvement in PA condition for the heterogeneous trials*
Kamijo et al. (2011)	Healthy children	RCT + pre/post - intervention	- FITTKIDS physical activity program - Wait-list control	8.9 9.1	12 (n/a) 14 (n/a)	600 p/w 36 weeks	Brain function (EEG)	- iCNV amplitude - tCNV amplitude *- Working memory RT (Sternberg task)* *- Working memory accuracy (Sternberg task)*	> Greater P3 amplitudes in the PA condition* > ns* > ns *> ns*
Kim and So (2015)	Healthy children	RCT + pre/post - intervention	- Combined exercise training (CET) - Sedentary control group	8.3 9.2	10 (40 %)10 (40 %)	180 p/w 16 weeks	Brain function (EEG)	- Alpha waves (Fp1, F3, F4, C4) *- Selective attention (Stroop task)*	> Greater amplitudes in the PA condition* > Greater improvement in PA condition
Krafft, et al. (2014c)^B^	Obese children	RCT + pre/post - intervention	- SMART physical activity program - Sedentary control group	9.5 9.6	13 (23 %)9 (44 %)	200 p/w 32 weeks	Brain function (resting state MRI)	- Resting-state fMRI: Salience network - Resting-state fMRI: Default mode network - Resting-state fMRI: Cognitive control network - Resting-state fMRI: Motor network *- Cognition (CAS)*	> ns > Decreased synchrony in PA condition > Decreased synchrony in PA condition > Increased synchrony in PA condition *> ns*
Krafft, et al. (2014c)^B^	Obese children	RCT + pre/post - intervention	- SMART physical activity program - Sedentary control group	9.7 9.9	24 (29 %)19 (42 %)	200 p/w 32 weeks	Brain function (MRI)	- Active-state fMRI (Antisaccade task) - Active-state fMRI (Flanker task) *- Inhibition RT (Antisaccade task)* *- Inhibition accuracy (Antisaccade task)* *- Attention inhibition RT (Flanker task)* *- Attention inhibition accuracy (Flanker task)*	> Decreased activation in PA condition in: bilateral precentral gyrus, MFG, paracentral lobule, postcentral gyrus, SPL, IPL and ACC > Increased activation in PA condition in: bilateral SFG, MFG, MFG, cingulate gyrus, and ACC *> ns* *> ns* *> ns* *> ns*
Krafft, et al. (2014b)^B^	Obese children	RCT + pre/post - intervention	- SMART physical activity program - Sedentary control group	9.9 9.4	10 (50 %)8 (50 %)	200 p/w 32 weeks	Brain structure WMI (MRI)	- WMI SLF FA - WMI SLF RD *- Cognitive function (CAS)* *- Teacher ratings of executive function behaviors (BRIEF)*	> Only when attendance was considered, increased WMI was observed in PA condition* > Only when attendance was considered, decreased WMI was observed in PA condition* > ns *> Only when attendance was considered, improved executive function was observed in PA condition*
Lee et al. (2017)	ADHD	RCT + pre/post - intervention	- Combined exercise group - Non exercise control group	8.8 8.8	6 (100 %)6 (100 %)	180 p/w 12 weeks	Brain function (EEG)	- Beta waves eyes open - Beta waves eyes closed - Beta waves (Stroop task) *- Selective attention (Stroop task)*	> ns* > ns* > Greater beta wave amplitudes in PA condition* > ns
Ludyga et al. (2019)	Healthy children	RCT + pre/post - intervention	- Aerobic training group - Coordinative training group - Control group	9.1 9.6 9.3	11 (n/a) 12 (n/a) 14 (n/a)	135 p/w 10 weeks	Brain function (EEG)	- P3 amplitude - P3 latency *- Attention inhibition RT (Flanker task)* *- Attention inhibition accuracy (Flanker task)*	> ns > ns *> ns* *> ns*
Schaeffer et al. (2014)^B^	Obese children	RCT + pre/post - intervention	- SMART physical activity program - Sedentary control group	9.9 9.4	10 (n/a) 8 (n/a)	200 p/w 32 weeks	Brain structure WMI (MRI)	- WMI uncinate fasciculus FA - WMI uncinate fasciculus RD	> Increased FA in the bilateral uncinate fasciculus in the PA condition* > Decrease RD in the left uncinate fasciculus in the PA condition*
Xiong et al. (2018)	Deaf children	RCT + pre/post - intervention	- Aerobic exercise program - Control group	10.2 11.5	10 (30 %)8 (50 %)	180 p/w 11 weeks	Brain structure WMI (MRI)	- WMI FA - WMI MD *- Inhibition RT (Flanker task)* *- Inhibition accuracy (Flanker task)* *- Working memory RT (2-back task)* *- Working memory accuracy (2-back task)* *- Shifting RT (odd task)* *- Shifting accuracy (odd task)*	> Decreased FA in the PA condition in: PCT, right HC, gCC, left SFOF, right ICP and left SCR* > Increased MD in the PA condition in: gCC, ALIC, IFOF, HC and decreased MD in: left ICP, left TAP* > Improved performance in PA condition *> ns* *> Decreased performance in PA condition* *> ns* *> Improved performance in PA condition* *> ns*

Note. * Measures are included in meta-analysis. The measures without an asterisk were not included in the meta-analysis because these studies did not report sufficient information to allow meta-analysis; Italicized measures are behavioral measures; ^A, B^ Studies used overlapping samples; ACC, anterior cingulate cortex; ALIC, anterior limb of the internal capsule; CAS, cognitive assessment system; FA, fractional anisotropy; fMRI: functional magnetic resonance imaging; FEF, frontal eye field; gCC, genu of the corpus callosum; HC, hippocampus; ICP, inferior cerebellar peduncle; IFOF, inferior frontooccipital fasciculus; IPL, inferior parietal lobule; MD, mean diffusivity; MRI: magnetic resonance imaging; n/a, not available; MFG, medial frontal gyrus; PA: Physical activity; PFC: prefrontal cortex; PCT, pontine crossing tract; PPC, posterior parietal cortex; RD, radial diffusivity; RT; reaction time; SCR, superior corona radiata; SEF, supplementary eye field; SFOF, superior frontooccipital fasciculus; SLF, superior longitudinal fasciculus; SFG, superior frontal gyrus; SPL, superior parietal lobule; TAP, tapetum; WMI, white matter integrity.

**Table 3:** Glossary and interpretation of the effect direction of imaging measures in meta-analysis.

EEG	
**Alpha waves**	Neural oscillations in the frequency range of 7.5–12.5 Hz, reflecting the resting state for the brain (relaxed awareness). Lower values are interpreted as abnormal (Huang et al., 2018).
**Beta waves**	Neural oscillations in the frequency range of 12.5−30 Hz, reflecting a state concentration and alertness. Lower values are interpreted as abnormal (Huang et al., 2018).
**Conflict sustained potential** **(Conflict SP)**	Relative slow ERP, which occurs approximately 500 ms. after stimulus onset, reflecting neural activity that response to the presence of a conflict or response selection. Higher values are interpreted as more positive (Larson et al., 2011).
**Cortical current density**	The electric current caused by neural activity per square millimetre, in which lower values are interpreted as more positive (Wollseiffen et al., 2018).
**Contingent negative variation (CNV)**	Slow negative ERP appearing during a reaction time task between a warning and an imperative stimulus, in which a greater CNV amplitude reflects a higher efficiency of stimuli processing (Grünewald-Zuberbier et al., 1978).
**Error related negativity (ERN)**	The response-locked negative deflection reflecting the response to an error of commission, which represents reinforcement learning of error detection. Lower amplitudes are interpreted as positive (Drollette et al., 2018).
**Initial CNV** **(iCNV or CNV 1)**	Early CNV wave reflecting orienting response to a stimulus and stimulus processing or evaluation. Greater amplitudes are interpreted as more positive (Loveless and Sanford, 1974).
**Terminal CNV** **(tCNV or CNV 2)**	Late CNV wave reflecting anticipatory attention for upcoming stimuli and motor preparation. Greater amplitudes are interpreted as more positive (Loveless and Sanford, 1974).
**Theta waves**	Neural oscillations in the frequency range of 4–8 Hz, reflecting meditative, drowsy and non-deep sleeping states. Higher values are interpreted as abnormal (Huang et al., 2018).
**Theta/Alpha ratio**	An index which shows the percentage of alpha versus theta waves. Higher values are interpreted as abnormal (Huang et al., 2018).
**Theta/Beta ratio**	An index which shows the percentage of beta versus theta waves. Higher values are interpreted as abnormal (Huang et al., 2018)
**P3 amplitude**	The magnitude of the P3 component, which appears approximately 300 ms after stimulus onset, reflecting the allocation of attentional resources toward the target stimulus. Higher values are interpreted as more positive (Polich, 2007).
**P3 latency**	The duration of the P3 component, which appears approximately 300 ms after stimulus onset, reflecting the processing time of the allocation of attentional resources toward the stimulus. Lower values are interpreted as more positive (Ila and Polich, 1999).
**P3a amplitude**	Positive waveform of the P3 component with short peak latency, reflecting orienting attention to novel stimuli. Higher values are interpreted as more positive (Polich, 2007).
**P3b amplitude**	Positive waveform of the P3 component during target stimulus processing, reflecting the allocation of attention during stimulus engagement. Higher values are interpreted as more positive (Polich, 2007).
**MRI**	
**Active-state fMRI**	MRI measurement that determines brain activity during cognitive tasks by detecting associated changes in BOLD signal. Changes in BOLD signal (either increased or decreased signal) are in this study interpreted as positive (Jansma et al., 2001).
**Fractional anisotropy (FA)**	DTI measurement that represents the degree to which diffusion is anisotropic, in which high FA values indicate that diffusion is greater in one direction that others, whereas low FA values indicate that diffusion is nearly equal in every direction. A high degree of myelination would cause axons to be tightly packed together and would leave less intercellular water than a low degree of myelination. Higher values are interpreted as more positive (Feldman et al., 2010).
**Axial diffusivity (AD)**	DTI measurement that represents the rate of diffusion in the direction that is parallel to the white matter tract. Higher values are interpreted as more restriction and less diffusion and are interpreted as positive (Feldman et al., 2010).
**Mean diffusivity (MD)**	DTI measurement that represents the net degree of displacement of the water molecules. Lower values are interpreted as more restriction and less diffusion and are interpreted as positive (Feldman et al., 2010).
**Radial diffusivity (RD)**	DTI measurement that represents the rate of diffusion in the direction that is perpendicular to the white matter tract. Lower values are interpreted as more restriction and less diffusion and are interpreted as positive (Feldman et al., 2010).
**Resting-state fMRI**	MRI measurement that determines brain activity during rest by detecting associated changes in the BOLD signal. Changes in BOLD signal (either increased or decreased signal) are in this study interpreted as positive (Jansma et al., 2001).

Abbreviations: EEG, Electroencephalogram; DTI, Diffusion Tensor Imaging; fMRI, functional magnetic resonance imaging; BOLD signal, Blood-oxygen-level dependent signal.

### 3.1 Acute effects of physical activity

No studies addressed the acute effects of physical activity on brain structure. One study addressed the acute effects of physical activity on cerebral blood flow (Pontifex et al., 2018). Results indicated no acute effects of physical activity in cerebral blood flow in the frontoparietal, executive control, and motor networks. Eleven cross-over studies addressed the acute effects on neurophysiological functioning, of which seven included healthy children (EEG; *k* = 6; MRI; *k* = 1). All studies in healthy children showed physical activity-induced acute effects on neurophysiological functioning. Results indicated improved neurophysiological functioning during rest (Mierau et al., 2014) and goal-directed behavior (Chen et al., 2016; Mierau et al., 2014; Wollseiffen et al., 2018), greater allocation of attentional resources during task performance (Chu et al., 2017; Hillman et al., 2009; Lind et al., 2019; St Louis Deschenes et al., 2015) and improved conflict processing (Chu et al., 2017). Three of the seven studies reported accompanying beneficial effects on measures of cognitive performance (Chen et al., 2016; Hillman et al., 2009; Lind et al., 2019) or academic functioning (Hillman et al., 2009).

Four studies addressed the acute effects of short-term physical activity on neurophysiological functioning in clinical samples (i.e. ADHD; EEG *k* = 4) and all these studies indicated physical activity-induced beneficial effects. Results indicate greater allocation of attentional resources toward the target stimulus (Hung et al., 2016; Pontifex et al., 2013), shorter processing time (Pontifex et al., 2013), improved anticipatory attention performance and motor preparation (Chuang et al., 2015) and an improved theta/beta ratio in resting EEG (Huang et al., 2018). Two studies reported on co-occurring beneficial cognitive effects and beneficial effects on academic performance (Chuang et al., 2015; Pontifex et al., 2013).

### 3.2 Chronic effects of physical activity

Four studies (MRI *k* = 4) described the chronic effects on brain structure in which one study included a healthy population and three studies included a clinical population (obesity *k =* 2; deafness *k* = 1). All studies used Diffusion Tensor Imaging, which is an MRI-based measure of white matter integrity (WMI). The study that assessed healthy children observed greater WMI in the genu of the corpus collosum following long-term physical activity compared to the control group (Chaddock-Heyman et al., 2018). The two studies assessing obese children observed greater WMI following long-term physical activity compared to a control group. None of the two studies reported concomitant objective cognitive measures. In contrast, a study in deaf children found decreased WMI following long-term physical activity (Xiong et al., 2018). The study also observed accompanying effects on measures of cognitive performance, of which some effects were beneficial, while others were detrimental.

Ten studies described the chronic effects on neurophysiological functioning of which six studies focused on healthy children (EEG; *k* = 5; MRI: *k* = 1). Five studies in healthy children showed physical activity-induced effects on neurophysiological functioning. Results indicated improved resting-state attention (Kim and So, 2015) and altered brain activation in the right anterior PFC (Chaddock-Heyman et al., 2013), improved error detection (Drollette et al., 2018), greater efficiency of attention and motor processes (Kamijo et al., 2011), greater allocation of attentional resources during goal-directed behavior and shorter processing time (Hillman et al., 2014). In addition, the observed changes in neurophysiological functioning were accompanied by improved cognitive task performance in all studies (Kamijo et al., 2011; Chaddock-Heyman et al., 2013; Hillman et al., 2014; Kim and So, 2015; Drollette et al., 2018).

Four studies described the chronic effects on neurophysiological functioning on clinical samples (EEG; *k* = 1; MRI; *k* = 3). One study investigated the chronic effects of physical activity in children with ADHD and found an improved state of alertness after long-term physical activity as measured by EEG. This result was not accompanied by improved cognitive task performance (Lee et al., 2017). All three studies investigating the chronic effects of long-term physical activity in obese children indicated changes in neurophysiological functioning as measured using fMRI. Results indicate altered brain activity during goal-direct behavior (Davis et al., 2011; Krafft et al., 2014c) and resting-state (Krafft et al., 2014a). None of these studies observed accompanying beneficial effects on cognitive task performance.

### 3.3 Risk of bias

Results of the risk of bias assessment, using the Cochrane Collaboration’s tool for risk of bias in randomized trials, are shown in Table 4. The overall risk of bias of the included studies varied, but was generally low. However, in only five studies outcome assessors were blinded (Chaddock-Heyman et al., 2018; Davis et al., 2011; Drollette et al., 2018; Hillman et al., 2014; Ludyga et al., 2019) and in ten studies the included population was not a representative sample for the general healthy or clinical pediatric population (Chu et al., 2017; Chuang et al., 2015; Huang et al., 2018; Hung et al., 2016; Krafft et al., 2014a; 2014b; 2014c; Lee et al., 2013; Mierau et al., 2014; Schaeffer et al., 2014). Five of the 26 studies (19 %) were preregistered in clinical trial registers. Of these studies, all reported outcome measures included in meta-analysis were preregistered. Conversely, not all preregistered outcome measures were reported in the available articles

**Table 4:** Risk of bias assessed by Cochrane Risk of Bias tool.

Studies	Sequence generation	Allocation concealment	Blinding participants	Blinding of outcome assessment	Incomplete outcome data	Selective outcome reporting	Sampling bias
Chaddock-Heyman et al. (2013)*	Unclear	Unclear	High	Unclear	Low	Low	Low
Chaddock-Heyman et al. (2018)*	Low	Low	High	Low Blinded research team	Low	Low	Low
Chen et al. (2016)	Unclear	N/A Cross-over design	High	Unclear	Unclear	Low	Low
Chu et al. (2017)	Unclear	N/A Cross-over design	High	Unclear	Unclear	Low	High 100 % boys
Chuang et al. (2015)	Unclear	N/A Cross-over design	High	Unclear	Low	Low	High 85 % boys
Davis et al. (2011)	Low Stratified	Low	High	Low Blinded assessors	Low	Unclear Not reported for subsample	Low
Drollette et al. (2018)*	Low Balanced by sex, race, IQ, SES & VO2; Coin flipping	Unclear	High	Low Blinded staff members	Low	Low	Low
Hillman et al. (2009)	Unclear	N/A Cross-over design	High	Unclear	Unclear	Low	Unclear
Hillman et al. (2014)*	Low Coin flipping	Low	High	Low Blinded assessors	Low	Low	Low
Huang et al. (2018)	Low Counterbalanced	Unclear	High	Unclear	Low	Low	High 100 % boys
Hung et al. (2016)	Unclear	N/A Cross-over design	High	Unclear	Low	Low	High 100 % boys
Kamijo et al. (2011)	Unclear	Unclear	High	Unclear	Low	Low	Low
Kim and So (2015)	Unclear	Unclear	High	Unclear	Low	Low	Low
Krafft, et al. (2014c)	Low Balanced by sex, race, school	Low	High	Unclear	Low	Low	High 100 % black
Krafft et al. (2014c)	Low Balanced by sex, race, school	Low	High	Unclear	Low	Low	High 90 % black
Krafft et al. (2014b)	Low Balanced by sex, race, school	Low	High	Unclear	Low	Low	High 94 % black
Lee et al. (2017)	Low Block randomization	Unclear	High	Unclear	Low	Low	High 100 % boys
Lind et al. (2019)	Low	N/A Cross-over design	High	Unclear	Low	Low	Low
Ludyga et al. (2019)*	Low	Unclear	High	Low Blinded assessors	Low	Low	Unclear
Mierau et al. (2014)	Unclear	N/A Cross-over design	High	Unclear	Low	Low	High 100 % boys
Pontifex et al. (2013)	Unclear	N/A Cross-over design	High	Unclear	Unclear	Low	High 70 % boys
Pontifex et al. (2018)	Low Counterbalanced	N/A Cross-over design	High	Unclear	Low	Low	Low
Schaeffer et al. (2014)	Unclear	Low	High	Unclear	Unclear	Low	High 95 % black
St Louis Deschenes et al. (2015)	Unclear	N/A Cross-over design	High	Unclear	Unclear	Low	Low
Xiong et al. (2018)	Unclear	Unclear	High	Unclear	Low	Low	Low
Wollseiffen et al. (2018)	Unclear	N/A Cross-over design	High	Unclear	Unclear	Low	Low

Abbreviations: N/A, not applicable; * Outcome measures were preregistered in clinical trial registers.

### 3.4 Meta-analysis

**Fig. 2:**
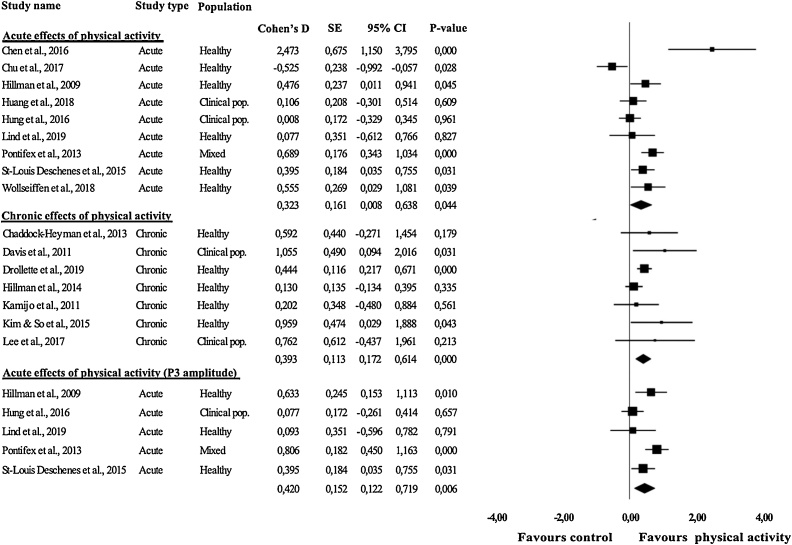
Effect sizes of studies concerning the effects of physical activity on neurophysiological functioning. Magnitude of symbols depicting the individual effect sizes is proportional to the number of subjects included in the study.

Results of the meta-analysis are displayed in Table 5 and Fig. 2. There were no studies available that investigated acute effects of physical activity on brain structure. Meta-analyses of studies which observed the acute effects of physical activity revealed a significant small-sized effect of physical activity on neurophysiological function (*d* = 0.32*, p* = 0.044). Further distinction between children from healthy samples and clinical samples revealed no significant meta-analytic effects in these subgroups.

**Table 5:** Overview of meta-analytic effect sizes.

	*n*	*k*	Cohen’s D	Cl-95 %	P -value	I^2^	I^2^ Cl-95%^†^	fsN	Egger p
**Acute effects of physical activity**									
Brain structure	–	–	–	–	–	–	–	–	–
Healthy	–	–	–	–	–	–	–	–	–
Clinical	–	–	–	–	–	–	–	–	–
Neurophysiological functioning	**244**	**9**	**0.32**	**0.00 – 0.64**	**0.044**	37.23	0 – 78.06	31	0.374
Healthy^‡^	146	6	0.40	−0.10 – 0.89	0.116	42.72	0 – 88.88	11	0.343
Clinical (ADHD only)^‡^	58	2	0.05	−0.21 – 0.31	0.716	0.00	n/a	n/a	n/a
**Chronic effects of physical activity**									
Brain structure	197	4	0.37	−0.33 – 1.07	0.305	68.59	0 – 72.33	1	0.812
Healthy	–	1	–	–	–	–	–	–	–
Clinical	54	3	0.43	−0.79 – 1.65	0.492	1.78	0 – 13.08	0	0.600
Neurophysiological function*	**629**	**7**	**0.39**	**0.17 – 0.61**	**0.000**	0.00	0 – 69.63	32	0.186
Healthy*	**598**	**5**	**0.35**	**0.12 – 0.56**	**0.002**	0.00	0 – 78.79	15	0.552
Clinical	**31**	**2**	**0.94**	**0.19 – 1.69**	**0.014**	0.00	n/a	n/a	n/a
**P3 amplitude**									
Acute effects*	**175**	**5**	**0.42**	**0.12 - 0.72**	**0.006**	0.00	0 – 78.35	21	0.910
Healthy*	**101**	**3**	**0.42**	**0.16 - 0.69**	**0.002**	0.00	0 – 76.35	4	0.457
**P3 latency**									
Acute effects	143	4	0.24	−0.09 – 0.57	0.148	0.00	0 – 83.81	2	0.838
Healthy	69	2	0.24	−0.14 – 0.62	0.208	0.00	n/a	n/a	n/a

Note. ^†^ Negative values were set to zero; ^‡^ One study with a mixed sample was excluded, because no data were available of the separate groups; *Effects remained significant after excluding the active-state fMRI studies (*n* = 3); CI, confidence interval; Egger p, p-value Egger Funnel plot; fsN, fail-safe N; *k*, number of studies; n, number of participants; n/a, not applicable.

No significant meta-analytic effects were found for the chronic effects of physical activity on brain structure. Meta-analyses of studies that observed the chronic effects of physical activity on neurophysiological function revealed a significant small-sized positive effect of physical activity (*d* = 0.39*, p* < 0.001).Analyses making a further distinction between children from healthy and clinical samples revealed a significant small effect for healthy children (*d* = 0.32*, p* = 0.002) and a large effect for children from clinical populations (*d* = 0.94*, p* = 0.014).

We estimated separate meta-analytic effects for specific outcome measures that were available in at least two studies. We found a significant small-sized effect of physical activity on P3 amplitude (*d* = 0.39, *p* = 0.001). When we made a distinction between acute and chronic effects, meta-analytic effects could only be determined for acute effects, for which a significant small-sized effect was found (*d* = 0.42, *p* = 0.006). In further distinctions between healthy and clinical samples, meta-analytic effect sizes could only be determined for healthy samples. Meta-analyses revealed significant small-sized effect on P3 amplitude for acute effects of physical activity for healthy samples (*d* = 0.42, *p* = 0.002). For P3 latency, meta-analytic effects could only be determined for all studies together, acute effects of physical activity and the acute effects of physical activity for healthy children. No significant effects were observed.

Lastly, we performed a sensitivity analysis to explore the possibility that our strategy to value the physical activity-induced changes in brain activation in the active-state fMRI studies as beneficial, influenced our results (see Appendix, Table A2). We reran all analyses after excluding the results of all three active-state fMRI studies (Chaddock-Heyman et al., 2013; Chen et al., 2016; Davis et al., 2011), replicating all the reported significant meta-analytic effects on neurophysiological functioning, with the exception of the effect size for the acute effects of physical activity on neurophysiological functioning, which did not reach significance in the sensitivity analysis (*d* = 0.23, *p* = 0.093). The sensitivity analysis could not be executed for the effect size describing chronic effects on neurophysiological functioning in clinical populations, because the number of available studies dropped below the minimum of two studies required for meta-analysis.

### 3.5 Heterogeneity, robustness & publication bias

The heterogeneity ranged from 0 to 69%, with only high values of heterogeneity for the meta-analysis effect describing the chronic effects of physical activity on brain structure. The leave-one-out analysis revealed that none of the individual studies had an extreme influence on the meta-analytic effect sizes (Fig. 1A, see Appendix) for acute effects of physical activity on neurophysiological functioning (range: d = .232–.416, p = .004–.119), chronic effects on neurophysiological function (range: d = .349–.489; p = .000–.013) and acute effects on P3 amplitude (range: d = .241–.490; p = .000–.073). These findings indicate stability of the meta-analytic effect size estimations. For the meta-analytic effect size of acute effects of physical activity on neurophysiological functioning, it should be noted that the p-values in the leave-one-out analysis did not consistently meet conventional levels of significance (p = .004–.119). This possibly reflects heterogeneity among study outcomes underlying the effect size, and warrants caution when interpreting this result.

P-curve analysis for all significant meta-analytic effects (studies concerning acute and chronic effects of physical activity on neurophysiological function and studies concerning acute effects using P3 amplitude) indicated the presence of evidential value (all tests for right skewness of half p-curves: *ps* < .01). P-curve analysis could not be executed for the significant chronic effects on neurophysiological function in clinical populations and for acute effects on P3 amplitude in healthy children, because the number of studies with significant effects was too low. See Appendix, Figs. A2–A5 for the p-curve plots. Analysis of Egger funnel plot asymmetry revealed no evidence for publication bias (*ps* = .19–.91). Fail-safe N values indicated that the reported meta-analytic effects were not robust against influence of publication bias. However, the number of positive findings is as expected from the power of the retrieved studies, reflecting no evidence of excess significance for all meta-analytic results (χ^2^ = 0.001–5.048, *ps* > 0.10).

## 4 Discussion

This study is the first systematic review and meta-analysis focusing on the causal effects of physical activity on brain structure and neurophysiological functioning in children from healthy and clinical samples. Based on 26 studies with RCT or crossover designs representing 973 unique children, the results provide evidence of physical activity-induced changes on neuroimaging measures and in particular small-sized beneficial effects of physical activity on neurophysiological functioning in children. These findings underline the importance of physical activity for brain development in children.

The current study differentiated between acute effects resulting from a single bout of physical activity (or short-term physical activity) and chronic effects resulting from longer periods of continuous physical activity (long-term physical activity). Meta-analysis revealed support for both acute and chronic effects on neurophysiological functioning, while no evidence for effects on brain structure was found. This observed discrepancy is primarily accounted for by a very limited number of studies (*k* = 4) that assessed the effects of physical activity on brain structure, that also assessed heterogeneous samples of healthy, obese and deaf children. It is unknown whether these groups respond comparably to physical activity. If sample-specific mechanisms may contribute the effects of physical activity, this may have contributed to heterogeneity in the combined effect size.

Analyses aimed at specific neuroimaging measures showed that acute effects of physical activity could primarily be driven by changes in the allocation of attentional resources (P3 amplitude), rather than changes in the processing time (P3 latency). This is in line with the results of a recent systematic review indicating that physical activity and cardiorespiratory fitness are associated with P3b modulation during cognitive control and attention tasks (Kao et al., 2019). Although this hypothesis awaits replication in future research, a specific effect of physical activity on attention resource allocation would be highly relevant when physical activity is considered as an intervention to promote cognitive functioning in diverse populations with poor attentional skills, such as otherwise normally developing children at school or clinical groups such as children suffering from ADHD.

The current review and meta-analysis made a distinction between studies in healthy and clinical samples of children. Although we found no evidence for differences between healthy and clinical populations in the effect magnitude of physical activity, the possibility exists that the dominant mechanisms of action underlying the effects of physical activity on brain structure and neurophysiological function (partly) depend on health status and on the pathophysiology of the disorders studied (Bruce et al., 2011; Claesdotter et al., 2018; Vysniauske et al., 2016; Yau et al., 2012). For example, it is suggested that physical activity might be a particularly powerful treatment of ADHD because it is supposed to upregulate dopamine and norepinephrine, two neurotransmitters that are both implicated in the pathophysiology of the disorder (Vysniauske et al., 2016). Interestingly, upregulation of dopamine and norepinephrine is also suggested to underlie the beneficial effects of stimulant medication used to treat ADHD and alleviating symptoms of the disorder (Ng et al., 2017). Likewise, vasoactive effects on cerebral arteries and neurotoxicity by hyperinsulinemia are suggested to play a crucial role in altered brain structure and function in people with obesity (Bruce et al., 2011; Raji et al., 2010) and may be counteracted by physical activity (Veronese et al., 2017). The current study does not allow to draw conclusions about effects of physical activity in clinical populations because of the heterogeneous pediatric populations studied (ADHD, obese and deaf children) and because studies into acute effects focused exclusively on children with ADHD whereas studies into chronic effects were primarily focused on obese children. To provide a better understanding of the potential of physical activity programs as a treatment approach in clinical populations, future studies should elucidate whether the effects of physical activity interact with health status and, more specifically, with the underlying pathophysiological processes that are supposed to be targeted by physical activity.

We have taken all efforts to carefully interpret the valence of meta-analytic effects, using a sequential decision chain for interpretation of the studies’ individual effect sizes. Nevertheless, the interpretation of changes in fMRI derived measures is a challenging matter (e.g. see de Wit et al., 2016). We performed a sensitivity analysis by repeating our analyses after excluding outcome measures with ambiguous interpretation (i.e. three fMRI studies). Only when the original findings were replicated by the sensitivity analysis, we also provided conclusions about the valence of the observed effects (i.e. whether it was beneficial or not). This sensitivity analysis replicated the meta-analytic effect concerning the chronic effect of physical activity on neurophysiological functioning, indicating that the evidence for neurophysiological changes in response to physical activity indeed involve beneficial effects. Not all meta-analytic results were replicated by sensitivity analysis. The acute effects of physical activity on neurophysiological function were no longer significant without the three active state fMRI studies. The difference between results of the original analysis and the sensitivity analysis can be explained by both a loss of statistical power and a meaningful difference in the effect size. In both cases, the results concerning the acute effects of physical activity, only support that physical activity may induce changes in neurophysiological functioning. The valence of these effects remains unknown. Nevertheless, in the context of the evident benefits of physical activity for the physical health (Álvarez-Bueno et al., 2017; de Greeff et al., 2018; Hillman et al., 2008; Penedo and Dahn, 2005; Warburton et al., 2006), it may be considered unlikely that any effects of physical activity would have detrimental effects on brain functioning. Future studies that include ambiguously interpretable outcome measures, such as active state fMRI studies, should include parallel cognitive assessment to allow clear interpretations about the valence of the observed effects.

The results of the systematic review provide an overview of all findings on cognitive functioning parallel to the observed changes in neural mechanisms. Almost all included studies in the systematic review reported on cognitive performance along with neuroimaging measures (22/26 studies; 85 %). Results showed that co-occurring improvement in at least one measurement of cognitive or academic performance was observed in half of the studies. More specifically, 55 % (6/11) of the studies that observed acute effects of physical activity on neurophysiological function and 42 % (5/12) of the studies that observed chronic effects on neurophysiological function reported co-occurring improvement. These percentages are in line with results of recent systematic reviews and meta-analyses concerning the effects of physical activity on cognition and academic performance in children, in which small to moderate effects were found (de Greeff et al., 2018; Singh et al., 2018; Verburgh et al., 2013). Another interesting finding is that only three studies reported significant associations between imaging and cognitive measures (Krafft et al., 2014b; 2014c; Xiong et al., 2018). One possible explanation for that neurophysiological effects are not systematically paralleled by behavioral improvement is the typical use of small-sized study samples in neuroimaging research, limiting the statistical power to reveal the pertinent associations. Otherwise, the relation between neurophysiological and behavioral effects of physical activity may be non-linear or a behavioral response to physical activity may not be detected until the neurophysiological response has reached a certain threshold level.

This systematic review and meta-analysis has some limitations. Some meta-analytic effect sizes were based on a relatively small numbers of studies, limiting statistical power and representativeness of evidence. Despite our effort to contact authors to provide additional information for inclusion in our meta-analysis, the proportion of missing data was relatively high (38 % of all outcome measures). It should also be noted that only five of the included studies (20 %) were preregistered trials. We used three different approaches to assess presence of and/or robustness to publication bias (Egger funnel plot asymmetry, the Rosenthal’s fail-safe n and the test of excessive significance). The findings indicate limited robustness of the reported effect sizes, but we did not find any evidence for the influence of publication bias on the meta-analytic findings. Nevertheless, the results warrant caution in the interpretation of the obtained effect sizes. However, although evidential value could not be analyzed in two effect sizes (i.e. chronic effects on neurophysiological function in clinical populations and acute effects on P3 amplitude in healthy children) and some instability has been noticed in the robustness of a single meta-analytic result (acute effects on neurophysiological functioning), comprehensive bias analysis on all other significant meta-analytic effects provided no lack of evidential value or limited robustness. Although the majority of studies (81 %) did not adopt proper procedures for blinding of intervention delivery and outcome assessment and almost half of the studies (42 %) included a sample that was not representative for the targeted population, the overall risk of bias was generally low among the included studies.

The current systematic review and meta-analysis shows that long-term physical activity leads to beneficial changes in neurophysiological function. In addition, short-term physical activity may induce changes in neurophysiological functioning, although this evidence showed limited robustness. Furthermore, there is preliminary evidence indicating that physical activity could be a useful intervention to promote neurophysiological functioning (and cognitive functioning) in diverse pediatric populations. However, more research is required to gain knowledge on the effects of physical activity in such specific populations. High-quality intervention studies should include both neuroimaging techniques and behavioral outcomes. Given the signs of limited robustness of the available evidence, future studies should also consider pre-registration to limit the influence of publication bias in this field. Nevertheless, to this date, the current study presents an overview of the best available evidence regarding the causal effects of physical activity on brain structure and neurophysiological functioning in children and underlines the importance of physical activity for brain development during childhood.

## Declaration of Competing Interest

All authors have no conflicts of interest relevant to this article to disclose.
