# Supplementary material for: The effects of physical activity on brain structure and neurophysiological functioning in children: A systematic review and meta-analysis
Source: Dev Cogn Neurosci. 2020 Jul 25;45:100828. doi: 10.1016/j.dcn.2020.100828 (PMC7451819; doi:10.1016/j.dcn.2020.100828)
Supplement: Supplementary file 1 [file mmc1.docx]

**7. APPENDIX**

A1. Search strings

**Pubmed**

(((("Magnetic Resonance Imaging"[Mesh] OR imaging[tiab] OR MRI[tiab] OR fMRI[tiab])

AND

("Brain"[Mesh] OR brain[tiab] OR cerebral[tiab])) OR "Diagnostic Techniques, Neurological"[Mesh:noexp] OR "Electroencephalography"[Mesh] OR "Neuroimaging"[Mesh:noexp] OR "Diffusion Tensor Imaging"[Mesh] OR "Functional Neuroimaging"[Mesh] OR "Evoked Potentials"[Mesh] OR electroencephalograph*[tiab] OR electro-encephalograph*[tiab] OR EEG[tiab] OR ERP[tiab] OR evoked potential*[tiab] OR event-related potential*[tiab] OR DTI[tiab] OR diffusion tensor imaging[tiab] OR tractograph*[tiab] OR neuroimaging[tiab] OR neuro-imaging[tiab] OR white matter[tiab] OR neuroradiograph*[tiab] OR neuro-radiograph*[tiab] OR P3[tiab] OR P3a[tiab] OR P3b[tiab] OR P300[tiab] OR brain wave*[tiab] OR brain activity[tiab] OR Alpha rhythm*[tiab] OR Alpha wave*[tiab] OR Beta rhythm*[tiab] OR Beta wave*[tiab] OR Alpha activity[tiab] OR Beta activity[tiab] OR resting state*[tiab] OR functional imaging[tiab] OR brain mapping[tiab] OR Contingent Negative Variation[tiab] OR connectom*[tiab])

AND

("Exercise"[Mesh] OR "Physical Education and Training"[Mesh] OR "Sports"[Mesh] OR motor activit*[tiab] OR physical activit*[tiab] OR locomotor activit*[tiab] OR exercis*[tiab] OR physical education[tiab] OR sport*[tiab] OR athletic*[tiab] OR athlete[tiab] OR athletes[tiab] OR walking[tiab] OR running[tiab] OR swimming[tiab] OR jogging[tiab])

AND

(child*[tw] OR schoolchild*[tw] OR infan*[tw] OR pediatri*[tw] OR paediatr*[tw] OR boy[tw] OR boys[tw] OR boyhood[tw] OR girl[tw] OR girls[tw] OR girlhood[tw] OR youth*[tw] OR preadolescent*[tw] OR pre-adolescent*[tw] OR youngster*[tw]))

AND

((randomized controlled trial[pt] OR controlled clinical trial[pt] OR randomized controlled trials[mh] OR random allocation[mh] OR double-blind method[mh] OR single-blind method[mh] OR clinical trial[pt] OR clinical trials[mh] OR "clinical trial"[tw] OR ((singl*[tw] OR doubl*[tw] OR trebl*[tw] OR tripl*[tw])

AND

(mask*[tw] OR blind*[tw])) OR "latin square"[tw] OR placebos[mh] OR placebo*[tw] OR random*[tw] OR research design[mh:noexp] OR comparative study[pt] OR evaluation studies[pt] OR follow-up studies[mh] OR prospective studies[mh] OR cross-over studies[mh] OR control[tw] OR controll*[tw] OR prospectiv*[tw] OR volunteer*[tw] OR compar*[tw]) NOT (animals[mh] NOT humans[mh]))

**Embase**

(((('nuclear magnetic resonance imaging'/exp OR imaging:ab,ti,kw OR mri:ab,ti,kw OR fmri:ab,ti,kw)

AND

('brain'/exp OR brain:ab,ti,kw OR cerebral:ab,ti,kw) OR 'neurologic examination'/de OR 'brain mapping'/exp OR 'connectome'/exp OR 'electroencephalography'/exp OR 'neuroimaging'/exp OR 'tractography'/exp OR 'brain electrophysiology'/exp OR 'evoked response'/exp OR 'resting state'/exp OR electroencephalograph*:ab,ti,kw OR 'electro-encephalograph*':ab,ti,kw OR eeg:ab,ti,kw OR erp:ab,ti,kw OR 'evoked potential*':ab,ti,kw OR 'event-related potential*':ab,ti,kw OR dti:ab,ti,kw OR 'diffusion tensor imaging':ab,ti,kw OR tractograph*:ab,ti,kw OR neuroimaging:ab,ti,kw OR 'neuro-imaging':ab,ti,kw OR 'white matter':ab,ti,kw OR neuroradiograph*:ab,ti,kw OR 'neuro-radiograph*':ab,ti,kw OR p3:ab,ti,kw OR p3a:ab,ti,kw OR p3b:ab,ti,kw OR p300:ab,ti,kw OR 'brain wave*':ab,ti,kw OR 'brain activity':ab,ti,kw OR 'alpha rhythm*':ab,ti,kw OR 'alpha wave*':ab,ti,kw OR 'beta rhythm*':ab,ti,kw OR 'beta wave*':ab,ti,kw OR 'alpha activity':ab,ti,kw OR 'beta activity':ab,ti,kw OR 'resting state*':ab,ti,kw OR 'functional imaging':ab,ti,kw OR 'brain mapping':ab,ti,kw OR 'contingent negative variation':ab,ti,kw OR connectom*:ab,ti,kw)

AND

('exercise'/exp OR 'physical activity'/exp OR 'training'/exp OR 'physical education'/exp OR 'sport'/exp OR 'motor activit*':ab,ti,kw OR 'physical activit*':ab,ti,kw OR 'locomotor activit*':ab,ti,kw OR exercis*:ab,ti,kw OR 'physical education':ab,ti,kw OR sport*:ab,ti,kw OR athletic*:ab,ti,kw OR athlete:ab,ti,kw OR athletes:ab,ti,kw OR walking:ab,ti,kw OR running:ab,ti,kw OR swimming:ab,ti,kw OR jogging:ab,ti,kw)

AND

('child'/exp OR child*:ab,ti,kw OR schoolchild*:ab,ti,kw OR infan*:ab,ti,kw OR pediatri*:ab,ti,kw OR paediatr*:ab,ti,kw OR boy:ab,ti,kw OR boys:ab,ti,kw OR boyhood:ab,ti,kw OR girl:ab,ti,kw OR girls:ab,ti,kw OR girlhood:ab,ti,kw OR youth*:ab,ti,kw OR preadolescent*:ab,ti,kw OR 'pre-adolescent*':ab,ti,kw OR youngster*:ab,ti,kw))

AND

(('clinical trial'/exp OR 'triple blind procedure'/exp OR 'double blind procedure'/exp OR 'single blind procedure'/exp OR 'randomization'/exp OR 'placebo'/exp OR 'methodology'/de OR 'comparative study'/de OR 'evaluation study'/de OR 'follow up'/exp OR 'prospective study'/exp OR 'crossover procedure'/exp OR 'clinical trial':ab,ti,kw OR ((singl*:ab,ti,kw OR doubl*:ab,ti,kw OR trebl*:ab,ti,kw OR tripl*:ab,ti,kw)

AND

(mask*:ab,ti,kw OR blind*:ab,ti,kw)) OR 'latin square':ab,ti,kw OR placebo*:ab,ti,kw OR random*:ab,ti,kw OR control:ab,ti,kw OR controll*:ab,ti,kw OR prospectiv*:ab,ti,kw OR volunteer*:ab,ti,kw OR compar*:ab,ti,kw) NOT ([animals]/lim NOT [humans]/lim))) NOT (conference*:it OR letter*:it OR editorial*:it)

**SportDiscus**

( ((DE "DIAGNOSTIC imaging" OR DE "MAGNETIC resonance imaging" OR TI (imaging OR MRI OR fMRI) OR AB (imaging OR MRI OR fMRI))

AND

(DE "BRAIN" OR DE "BRAIN stem" OR DE "CEREBELLUM" OR DE "CEREBRAL hemispheres" OR DE "MIND & body" OR DE "MOTOR neurons" OR TI (brain OR cerebral) OR AB (brain OR cerebral)) OR DE "ELECTROENCEPHALOGRAPHY" OR DE "BRAIN waves" OR DE "EVOKED potentials (Electrophysiology)" OR TI (electroencephalograph* OR "electro-encephalograph*" OR EEG OR ERP OR "evoked potential*" OR "event-related potential*" OR DTI OR "diffusion tensor imaging" OR tractograph* OR neuroimaging OR "neuro-imaging" OR "white matter" OR neuroradiograph* OR "neuro-radiograph*" OR P3 OR P3a OR P3b OR P300 OR "brain wave*" OR "brain activity" OR "Alpha rhythm*" OR "Alpha wave*" OR "Beta rhythm*" OR "Beta wave*" OR "Alpha activity" OR "Beta activity" OR "resting state*" OR "functional imaging" OR "brain mapping" OR "Contingent Negative Variation" OR connectom*) OR AB (electroencephalograph* OR "electro-encephalograph*" OR EEG OR ERP OR "evoked potential*" OR "event-related potential*" OR DTI OR "diffusion tensor imaging" OR tractograph* OR neuroimaging OR "neuro-imaging" OR "white matter" OR neuroradiograph* OR "neuro-radiograph*" OR P3 OR P3a OR P3b OR P300 OR "brain wave*" OR "brain activity" OR "Alpha rhythm*" OR "Alpha wave*" OR "Beta rhythm*" OR "Beta wave*" OR "Alpha activity" OR "Beta activity" OR "resting state*" OR "functional imaging" OR "brain mapping" OR "Contingent Negative Variation" OR connectom*) OR KW (electroencephalograph* OR "electro-encephalograph*" OR EEG OR ERP OR "evoked potential*" OR "event-related potential*" OR DTI OR "diffusion tensor imaging" OR tractograph* OR neuroimaging OR "neuro-imaging" OR "white matter" OR neuroradiograph* OR "neuro-radiograph*" OR P3 OR P3a OR P3b OR P300 OR "brain wave*" OR "brain activity" OR "Alpha rhythm*" OR "Alpha wave*" OR "Beta rhythm*" OR "Beta wave*" OR "Alpha activity" OR "Beta activity" OR "resting state*" OR "functional imaging" OR "brain mapping" OR "Contingent Negative Variation" OR connectom*) )

AND

( DE "EXERCISE" OR DE "AEROBIC exercises" OR DE "ANAEROBIC exercises" OR DE "CIRCUIT training" OR DE "EXERCISE for children" OR DE "EXERCISE for girls" OR DE "EXERCISE for youth" OR DE "EXERCISE therapy" OR DE "MUSCLE strength" OR DE "RUNNING" OR DE "SCHOOL exercises & recreations" OR DE "STRENGTH training" OR DE "PHYSICAL activity" OR DE "PHYSICAL fitness for children" OR DE "PHYSICAL education" OR DE "TRAINING" OR DE "SWIMMING" OR DE "WALKING" OR DE "RUNNING" OR DE "JOGGING" OR DE "RUNNING for children" OR DE "SWIMMING for children" OR DE "SWIMMING for infants" OR DE "SPORTS" OR DE "SCHOOL sports" OR DE "SPORTS for children" OR DE "SPORTS for girls" OR DE "SPORTS for youth" OR TI ("motor activit*" OR "physical activit*" OR "locomotor activit*" OR exercis* OR "physical education" OR sport* OR athletic* OR athlete OR athletes OR walking OR running OR swimming OR jogging) OR AB ("motor activit*" OR "physical activit*" OR "locomotor activit*" OR exercis* OR "physical education" OR sport* OR athletic* OR athlete OR athletes OR walking OR running OR swimming OR jogging) OR KW ("motor activit*" OR "physical activit*" OR "locomotor activit*" OR exercis* OR "physical education" OR sport* OR athletic* OR athlete OR athletes OR walking OR running OR swimming OR jogging) )

AND

( DE "CHILDREN" OR DE "BOYS" OR DE "GIRLS" OR DE "SCHOOL children" OR TI (child* OR schoolchild* OR infan* OR pediatri* OR paediatr* OR boy OR boys OR boyhood OR girl OR girls OR girlhood OR youth* OR preadolescent* OR "pre-adolescent*" OR youngster*) OR AB (child* OR schoolchild* OR infan* OR pediatri* OR paediatr* OR boy OR boys OR boyhood OR girl OR girls OR girlhood OR youth* OR preadolescent* OR "pre-adolescent*" OR youngster*) OR KW (child* OR schoolchild* OR infan* OR pediatri* OR paediatr* OR boy OR boys OR boyhood OR girl OR girls OR girlhood OR youth* OR preadolescent* OR "pre-adolescent*" OR youngster*) )

**Cochrane Reviews**

((((imaging OR MRI OR fMRI)

AND

(brain OR cerebral)) OR electroencephalograph* OR (electro NEXT encephalograph*) OR EEG OR ERP OR (evoked NEXT potential*) OR (event NEXT related NEXT potential*) OR DTI OR (diffusion NEXT tensor NEXT imaging) OR tractograph* OR neuroimaging OR (neuro NEXT imaging) OR (white NEXT matter) OR neuroradiograph* OR (neuro NEXT radiograph*) OR P3 OR P3a OR P3b OR P300 OR (brain NEXT wave*) OR (brain NEXT activity) OR (Alpha NEXT rhythm*) OR (Alpha NEXT wave*) OR (Beta NEXT rhythm*) OR (Beta NEXT wave*) OR (Alpha NEXT activity) OR (Beta NEXT activity) OR (resting NEXT state*) OR (functional NEXT imaging) OR (brain NEXT mapping) OR (Contingent NEXT Negative NEXT Variation) OR connectom*)

AND

((motor NEXT activit*) OR (physical NEXT activit*) OR (locomotor NEXT activit*) OR exercis* OR (physical NEXT education) OR sport* OR athletic* OR athlete OR athletes OR walking OR running OR swimming OR jogging)

AND

(child* OR schoolchild* OR infan* OR pediatri* OR paediatr* OR boy OR boys OR boyhood OR girl OR girls OR girlhood OR youth* OR preadolescent* OR (pre NEXT adolescent*) OR youngster*)):ab,ti,kw

**Table A2. Meta-analytic effect sizes without fMRI studies**

|  | *n* | *k* | Cohen’s D | Cl-95% | P -value | I^2^ | I^2^ Cl-95%^†^ | fsN | Egger p |  |
| --- | --- | --- | --- | --- | --- | --- | --- | --- | --- | --- |
| Acute effects of physical activity | | | | | | | | | |  |
| Brain structure | - | - | - | - | - | - | - | - | - |  |
| Healthy | - | - | - | - | - | - | - | - | - |  |
| Clinical | - | - | - | - | - | - | - | - | - |  |
| Neurophysiological functioning | 235 | 8 | 0.23 | -0.09 – 0.50 | 0.093 | 0.00 | 0 – 99.97 | 12 | 0.669 |  |
| Healthy | 137 | 5 | 0.20 | -0.20 – 0.60 | 0.321 | 0.00 | 0 – 79.74 | 0 | 0.785 |  |
| Clinical (ADHD only) | 58 | 2 | 0.05 | -0.21 – 0.31 | 0.716 | 0.00 | n/a | n/a | n/a |  |
| Chronic effects of physical activity | | | | | | | | | |  |
| Brain structure | 197 | 4 | 0.37 | -0.33 – 1.07 | 0.303 | 68.59 | 0 – 72.33 | 1 | 0.827 |  |
| Healthy | - | 1 | - | - | - | - | - | - | - |  |
| Clinical | 54 | 3 | 0.43 | -0.79 – 1.65 | 0.492 | 1.78 | 0 – 13.08 | 0 | 0.600 |  |
| Neurophysiological function | **587** | **5** | **0.34** | **0.11 – 0.57** | **0.003** | 0.00 | 0 – 78.92 | 15 | 0.519 |  |
| Healthy | **575** | **4** | **0.34** | **0.08 – 0.58** | **0.010** | 0.00 | 0 – 86.86 | 11 | 0.731 |  |
| Clinical | - | **1** | - | - | - | - | - | - | - |  |
| P3 amplitude | | | | | | | | | |  |
| Acute effects | **175** | **5** | **0.42** | **0.12 - 0.72** | **0.006** | 0.00 | 0 – 78.35 | 21 | 0.910 |  |
| Healthy | **101** | **3** | **0.42** | **0.16 - 0.69** | **0.002** | 0.00 | 0 – 76.35 | 4 | 0.457 |  |
| P3 latency | | | | | | | | | |  |
| Acute effects | 143 | 4 | 0.24 | -0.09 – 0.57 | 0.148 | 0.00 | 0 – 83.81 | 2 | 0.838 |  |
| Healthy | 69 | 2 | 0.24 | -0.14 – 0.62 | 0.208 | 0.00 | n/a | n/a | n/a |  |

Note. ^†^ Negative values were set to zero;; ^±^ Effect sizes that are characterized as robust are displayed in bold; CI, confidence interval; Egger p, p-value Egger Funnel plot; fsN, fail-safe N; *k*, number of studies; n, number of participants; N/A, not applicable


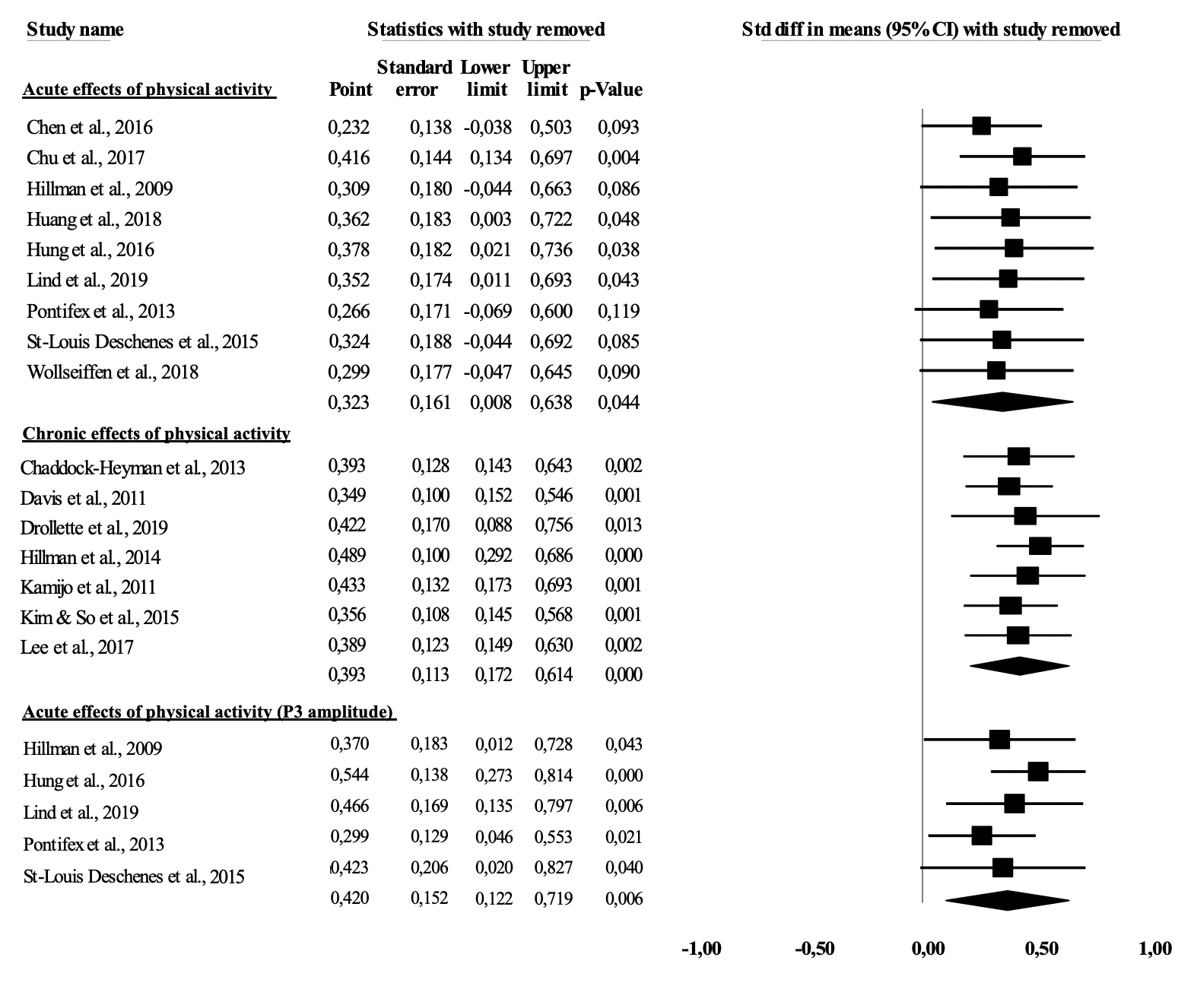


**Figure A1 Leave-one-out analysis**

**
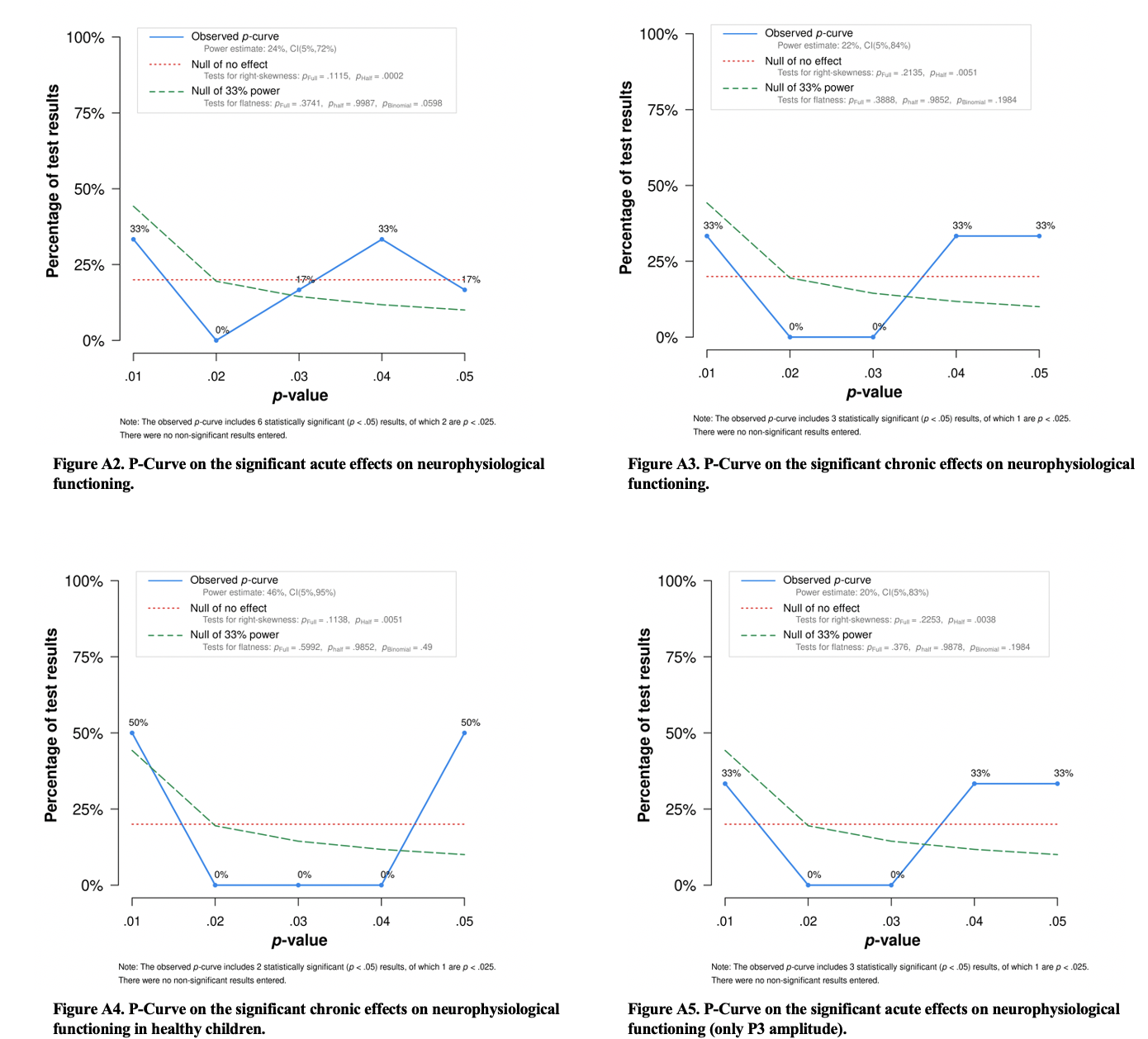
**
